# An empirical comparison of different approaches for combining multimodal neuroimaging data with support vector machine

**DOI:** 10.3389/fnins.2014.00189

**Published:** 2014-07-15

**Authors:** William Pettersson-Yeo, Stefania Benetti, Andre F. Marquand, Richard Joules, Marco Catani, Steve C. R. Williams, Paul Allen, Philip McGuire, Andrea Mechelli

**Affiliations:** ^1^Department of Psychosis Studies, Institute of Psychiatry, King's College LondonLondon, UK; ^2^Center for Mind/Brain Studies (CIMeC), University of TrentoTrento, Italy; ^3^Department of Neuroimaging, Centre for Neuroimaging Sciences, Institute of Psychiatry, King's College LondonLondon, UK; ^4^Department of Forensic and Neurodevelopmental Science, Institute of Psychiatry, King's College LondonLondon, UK

**Keywords:** support vector machine, integration, MRI, multi-kernel learning, label fusion, psychosis

## Abstract

In the pursuit of clinical utility, neuroimaging researchers of psychiatric and neurological illness are increasingly using analyses, such as support vector machine, that allow inference at the single-subject level. Recent studies employing single-modality data, however, suggest that classification accuracies must be improved for such utility to be realized. One possible solution is to integrate different data types to provide a single combined output classification; either by generating a single decision function based on an integrated kernel matrix, or, by creating an ensemble of multiple single modality classifiers and integrating their predictions. Here, we describe four integrative approaches: (1) an un-weighted sum of kernels, (2) multi-kernel learning, (3) prediction averaging, and (4) majority voting, and compare their ability to enhance classification accuracy relative to the best single-modality classification accuracy. We achieve this by integrating structural, functional, and diffusion tensor magnetic resonance imaging data, in order to compare ultra-high risk (*n* = 19), first episode psychosis (*n* = 19) and healthy control subjects (*n* = 23). Our results show that (i) whilst integration can enhance classification accuracy by up to 13%, the frequency of such instances may be limited, (ii) where classification can be enhanced, simple methods may yield greater increases relative to more computationally complex alternatives, and, (iii) the potential for classification enhancement is highly influenced by the specific diagnostic comparison under consideration. In conclusion, our findings suggest that for moderately sized clinical neuroimaging datasets, combining different imaging modalities in a data-driven manner is no “magic bullet” for increasing classification accuracy. However, it remains possible that this conclusion is dependent on the use of neuroimaging modalities that had little, or no, complementary information to offer one another, and that the integration of more diverse types of data would have produced greater classification enhancement. We suggest that future studies ideally examine a greater variety of data types (e.g., genetic, cognitive, and neuroimaging) in order to identify the data types and combinations optimally suited to the classification of early stage psychosis.

## Introduction

In response to growing demand for clinically translatable research (Matthews et al., 2006; Borgwardt and Fusar-Poli, 2012) neuroimaging investigators of psychiatric and neurological illness are increasingly using analyses that allow inference at the single subject level (Orrù et al., 2012). One such method is the support vector machine (SVM) classifier, which is able to classify individuals into predefined groups, and yield an associated accuracy indicative of how well it will generalize to future individual cases. A type of multivariate supervised pattern recognition algorithm, the use of SVM has become progressively widespread in both neurology and psychiatry to reveal patterns of alteration in patients relative to HCs that may potentially be used to (i) inform clinical diagnosis, and/or, (ii) predict treatment response (Orrù et al., 2012). When considering the ultimate development of SVM as a real-world clinical aid, however, arguably greater levels of discriminative accuracy are required than those currently reported. One method proposed to achieve this is the integration of data from different modalities, such that, complementary information from each modality can be used (Kittler et al., 1998). This is based on the premise that algorithms generated using different types of data will base their classifications on distinct patterns of alteration and also make distinct pattern misclassifications. Combining different classifiers within a single SVM therefore, or, alternatively, by creating an ensemble of multiple single modality SVMs, both aim to increase accuracy through the derivation of a consensus decision, as opposed to a single modality, single decision, classifier (Kittler et al., 1998). To date, existing applications involving Alzheimer's patients have generally shown encouraging, albeit modest, increases in predictive averaging ranging between 3 and 7% relative to the best single modality classification accuracy (BSMCA) (Fan et al., 2008; Hinrichs et al., 2011; Zhang et al., 2011). With specific reference to psychosis in comparison, only one recent study investigating ChSz has been published, in which the authors reported that using an integrative approach they were able to classify patients from HCs with 87.25% accuracy (Yang et al., 2010) representing an increase of approximately 5% relative to the BSMCA. Despite these promising results, these four studies employed only two methods, or variations thereof, for integrating data within SVM, namely, majority voting and multi kernel learning. Though alternative methods are available, to date no systematic investigation has yet been conducted examining the relative efficacies of a range of distinct integrative methods to combine multimodal neuroimaging data within the same clinical sample. It therefore remains unclear the extent to which combining data modalities can improve accuracy in a typical neuroimaging sample, and if so, which integrative approach provides the greatest classification increase and in what context.

In the current investigation, we provide a brief review of four different approaches that can be used to integrate data from multiple sources, namely, (1) an un-weighted “simple” sum of kernels (SK), (2) multi-kernel learning (MKL), (3) prediction averaging (AV), and (4) majority voting (MV). These particular methods were chosen on the basis that they: (i) are frequently used in the (limited) psychiatric and neurological literature (Fan et al., 2008; Yang et al., 2010; Hinrichs et al., 2011; Zhang et al., 2011) and/or (ii) are relatively straightforward to implement. We then apply each approach to the same data set in order to empirically examine their potential to enhance classification accuracy relative to the BSMCA. In addition, in order to investigate the impact made by the number of data types being combined on levels of integrated accuracy, we performed multi-modal integration using varying numbers of data types.

The data set to which each integrative method was applied is taken from work conducted recently by our own group in which we assessed the ability of different modalities to successfully classify first episode psychosis (FEP) and ultra-high risk (UHR) subjects from healthy controls (HCs), and from each other. For this study ethics approval was granted by the local Research Ethics Committee (reference number: 08/H0805/64). Our results showed that in conjunction with SVM, structural MRI (sMRI) data was able to discriminate UHR from HCs and FEP subjects with significant (*p* < 0.05) accuracies of 68.42 and 76.67%, respectively; diffusion tensor imaging (DTI) data was able discriminate both UHR and FEP from HCs with 65.79% accuracy; and, functional (MRI) data was able to discriminate FEP subjects from UHR and HCs with up to 68.42 and 73.33% accuracy, respectively (Pettersson-Yeo et al., 2013) (see Table 1). Based on these data, our primary aim was to examine the ability of the four integrative methods outlined, to enhance classification accuracy by integrating information from the three distinct neuroimaging modalities; sMRI, DTI, and fMRI (comprising one of two functional contrasts), in order to discriminate UHR from HCs, FEP from HCs, or UHR from FEP subjects, relative to the BSMCA for each diagnostic comparison.

**Table 1:** **SVM classification accuracies using single modality data for each diagnostic comparison**.

**SVM comparison**	**sMRI**	**DTI**	**fMRI contrast 1**	**fMRI contrast 2**	**fMRI contrast 3**	**fMRI contrast 4**	**fMRI contrast 5**
	**GM**	**FAS**	**In > CFI**	**Su > CFS**	**In > RI**	**Su > RS**	**Su > In**
UHR vs. HC (%)	68.42	65.79	36.84	60.53	57.89	60.53	47.37
	(68.42/68.42)	(68.42/63.16)	(36.84/36.84)	(57.89/63.16)	(57.89/57.89)	(57.89/57.89)	(31.58/63.16)
FEP vs. HC (%)	63.16	65.79	68.42	47.37	65.79	44.74	63.16
	(57.89/68.42)	(68.42/63.16)	(63.16/73.68)	(42.11/52.63)	(63.16/68.42)	(36.84/52.63)	(47.37/78.95)
FEP vs. UHR (%)	76.67	56.67	73.33	53.33	63.33	46.67	53.33
	(80.00/73.33)	(46.67/66.67)	(66.67/80.00)	(40.00/66.67)	(53.33/73.33)	(46.67/46.67)	(40.00/66.67)

*MRI, magnetic resonance imaging; sMRI, structural MRI; DTI, diffusion tensor imaging; fMRI, functional MRI; GM, grey matter; FAS, fractional anisotropy skeleton; In, generation of an overt verbal initiation response; Su, generation of an overt verbal suppression response; RI, repetition of “REST” during the initiation condition; RS, repetition of “REST” during the suppression condition; CFI, visual cross-fixation during the initiation condition; CFS, visual cross-fixation during the suppression condition; UHR, Ultra-High Risk; FEP, First Episode Psychosis; HC, Healthy Subjects. Figures in brackets are the sensitivity/specificity for each classifier*.

Since only a few studies have applied integrative techniques to neuroimaging data, for the purpose of the current study our hypotheses regarding which method may work best were informed from similar work in the field of proteomics (Lewis et al., 2006). Based on this previous work conducted by Lewis et al., in which alternative SVM integration methods were applied to the prediction of protein interactions and subsequently compared (Lewis et al., 2006), we hypothesized that (i) for two modality combinations, an un-weighted SK would perform as well, if not better, than the more sophisticated, weighted MKL, with AV likely to perform equally as well as MKL, (ii) for three modality combinations, MKL and AV would perform as well, or better than, SK and MV, given their respective ability to explicitly, or implicitly, dampen the contribution of “noisy” data to the definition of the optimal separating hyperplane (OSH), and (iii) based on the spectrum of different results obtained using single modality data in conjunction with SVM for each of the three diagnostic comparisons, the ability of each integrative method to enhance classification accuracy would vary depending on the diagnostic comparison to which it was applied.

## Materials and methods

### SVM

Originally developed in the early 1990s (Cortes and Vapnik, 1995), and stemming from statistical learning theory (Vapnik, 1999), SVM is a multivariate pattern recognition algorithm well suited to binary group classification. The SVM aims to learn a decision function that correctly predicts the class label (conventionally denoted by *y* = +1 or −1) for each data point, based on a set of *m* training examples {***x**_i_, y_i_*}^*m*^_*i* = 1_, where ***x**_i_* are data vectors associated with each label. The goal is then to predict the labels for a set of unseen testing examples (Burges, 1998). Under the linear kernel formulation employed in the present work, a dot product similarity measure was used to represent data in a symmetric, positive definite kernel matrix. In this feature space SVM can be used to linearly separate groups (i.e., classes) of individuals (e.g., FEP and UHR subjects). The linear SVM decision function (Equation 1) can be written as the dot product between each data vector and a vector of predictive weights (***w***). The predicted class label can then be derived by taking the sign of the decision function. The weight vector represents an OSH in the input (i.e., voxel) space and can be represented in terms of the most difficult data points to classify (referred to as support vectors). The optimal weight vector is determined by maximizing the margin between groups thus aiming to ensure good generalization to new data, an approximately unbiased estimate of which can be obtained using cross-validation (Hastie et al., 2001; Lemm et al., 2011). Here, leave-one-out cross validation (LOOCV) was employed, an iterative process whereby each subject is omitted during the training of the classifier and used as an independent test set to test the trained classifier's accuracy, with the final reported classification accuracy (i.e., proportion of subjects correctly classified) representing the average over all iterations. Whilst providing an approximately unbiased estimate of generalizability for a given sample, however, we note that this technique does not necessarily offset the impact of using a relatively small sample size in the context of generalization to as yet unseen psychosis subjects, with larger samples ultimately being the ideal. The SVM objective function is provided in Equations 2 and 3, reflecting the primal, and dual, space representations respectively. Here, ***w*** is a vector of predictive weights in the input (primal) space, *b* denotes offset, ε_*i*_ denote slack variables which permit data to be misclassified in the training set, α_*i*_ denote Lagrange multipliers (or dual space weights) and *C* is a parameter regulating the balance between maximizing the margin between data points and allowing misclassification in the training set. For a more detailed description of SVM see Burges (1998) or Schölkopf and Smola (2002). For an overview of SVM in the context of neuroimaging, see Pereira et al. (2009) and Lemm et al. (2011).

(1) $$f\text{​}\left(x,w\right)=w^{T}x+b$$

(2) $$\begin{array}{l} \begin{array}{ll} \text{minimize } & \frac{1}{2}{\left\|w\right\|}^{2}+C\sum_{\;i\;=\;1}^{m}\xi_{i} \end{array}\\ \text{subject to}:\;y_{i}\left(w^{T}x_{i}\right)\geq 1-\xi_{i}\\ \;\xi_{i}\geq 0\;\forall i \end{array}$$

(3) $$\begin{array}{l} \;\begin{array}{ll} \text{minimize} & \sum_{i=1}^{m}\alpha_{i}-\frac{1}{2}\sum_{i=1}^{m}\alpha_{i}\alpha_{j}y_{i}y_{j}k(x_{i},x_{j}) \end{array}\;\\ \begin{array}{ll} \text{subject to}: & 0\leq \alpha_{i}\leq C\;\forall i \end{array}\\ \;\sum_{i=1}^{m}y_{i}\alpha_{i}=0\;\forall i\; \end{array}$$

where *k*(***x***_*i*_, ***x***_*j*_) is the kernel, here taken to be a (linear) dot product between data samples. Equations 2 and 3 are convex optimization problems and can be efficiently optimized with conventional quadratic solvers. In the present work, the LIBSVM implementation was employed (Chang and Lin, 2011) as implemented in the PROBID software toolbox (http://www.brainmap.co.uk/probid.htm). As is common in neuroimaging data, the value of the SVM regularization parameter *C* was fixed to one.

### Combining classifiers

**Figure 1:**
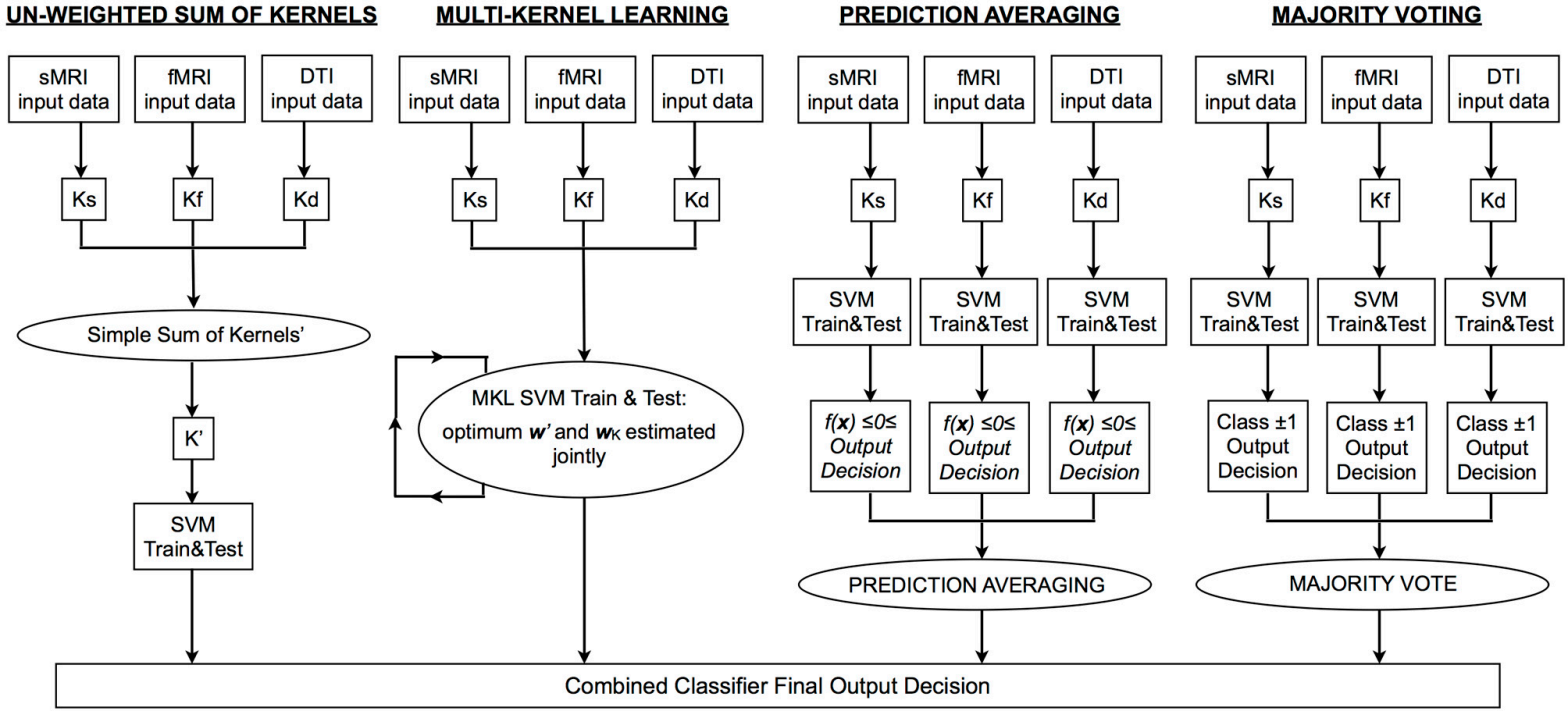
**Flowchart depicting the processing pipeline for each type of integrative approach**. MRI, magnetic resonance imaging; sMRI, structural MRI; DTI, diffusion tensor imaging; fMRI, functional MRI; Ks/f/d, kernel matrix for sMRI/fMRI/DTI data; SVM, support vector machine; K′, integrated kernel matrix; *f*(x), SVM decision function; ***w***′, optimum vector of predictive weights obtained using K′; ***w***_*K*_, optimum weight coefficient assigned to each base kernel.

In order to generate a single output decision from multiple sources, two options are, (1) find a linear combination of the kernel matrices representing each data modality in order to train and test a single SVM; in this case, predictive weights are estimated jointly from all data, or (2) train multiple single modality classifiers and subsequently combine the output decisions to generate a single decision function using label fusion techniques; in this case, the weight vectors for each data type are estimated independently. Of the four approaches used in the current investigation, SK and MKL are variations of option 1, and MV and prediction averaging are variations of option 2. In Figure 1 we depict a representative pipeline showing the steps used for each approach. For all classifiers, classification accuracy can be calculated by dividing the number of correct predictions by the total number of predictions. A balanced accuracy for two groups can be obtained based on the mean of the classifier's sensitivity and specificity; note that this is equivalent to the standard definition of accuracy (i.e., [True Positives + True Negatives]/[Total Number of Subjects]) when equal sized groups are used, as is the case here.

#### Un-weighted simple sum of kernels

A well-known property of kernels is that they can be combined via linear operations (e.g., addition and multiplication) to yield a valid kernel. As described, a linear kernel matrix is used in the present work to represent the similarity between data points within each data modality (Equation 4). Thus, a simple way to combine data modalities is simply to add the kernel matrices. Importantly, different modalities may have different numbers of features and may also be scaled differently. To account for this, each kernel was first normalized before being summed together to create a new kernel matrix representing data from all modalities (Equation 4: example shown represents combining two data types only, i.e., ***K*_1_** and ***K*_2_**. This is equivalent to first dividing each data vector by its Euclidean norm, then concatenating the feature vectors for all modalities. Under this framework, the data from each source are assigned an equal weighting in terms of their contribution in defining the OSH. A SVM is then trained and tested (Equations 2 and 3) using this new integrated kernel (***K***′), such that classification is based on all data sources.

(4) $$K^{\prime }=\frac{{\left(K_{1}\right)}_{ij}}{\sqrt{{\left(K_{1}\right)}_{ii}{\left(K_{1}\right)}_{jj}}}+\frac{{\left(K_{2}\right)}_{ij}}{\sqrt{{\left(K_{2}\right)}_{ii}{\left(K_{2}\right)}_{jj}}}$$

#### Multi-kernel learning

The MKL approach provides a means of automatic kernel combination with the aim of producing a “best kernel” from a linear combination of *q* input (“base”) kernels, such that the optimal kernel is given by:

$$K=\sum_{k=1}^{q}\beta_{k}K_{k}$$

where β_*k*_ are predictive weights for each base kernel. These are optimized simultaneously with the dual predictive weights in the ordinary SVM framework. Many different optimization and regularization frameworks exist for MKL (e.g., Lanckriet et al., 2004; Sonnenburg et al., 2006). In this work, we employed an MKL formulation based on elastic net regularization. See Tomioka and Suzuki (2010) for details. Under this framework, the regularization penalty of the model is a combination of L1 and L2 components. A tuning parameter λ ∈ [0, 1] governs the relative contribution of the respective L1 and L2 regularization terms, such that λ = 0 denotes an extremely sparse (L1) model, where many kernel weighting coefficients are pushed to zero and λ = 1 denotes a uniform weighted combination of kernels. The elastic net regularizer thus aims to find an optimal balance between enforcing sparsity and allowing kernels that are correlated with one another to participate in the model. In this work, we employ the implementation provided in the SHOGUN toolbox, (Sonnenburg et al., 2010; http://www.raetschlab.org/suppl/shogun). To ensure optimal performance in MKL, proper tuning of the elastic net regularization parameters (*C* and λ) is crucial. Here this was achieved using a nested cross-validated grid search, where *C* took the range of values from 0.001 to 1000 (six steps, iteratively increasing order of magnitude) Lambda, 0.1 to 1 in steps of 0.1.

#### Averaging

In contrast to SK, or MKL, AV integrates modalities at the level of predictions (i.e., forming an ensemble decision after each base classifier has been trained and tested on a single modality; see Figure 1). Integration is achieved by taking the mean of the predictive function values over all modalities and computing its sign to derive an average class prediction. Hence for a given subject (*i*), a base classifier is trained for each modality (Equation 3), which we denote by ***f(x,w_c_)***, *c* = 1, …, *q*, and the final class based on integrated data using AV predicted by:

$$y=sgn\left(\frac{1}{q}\sum_{c=1}^{q}f(x,w_{c})\right)$$

#### Majority voting

Similar to AV, MV also performs integration at the level of the predictions. However, for MV only the sign (i.e., binary outcome) of the decision function is considered, rather than its sign and magnitude as in AV. Under the MV approach, the final class label is therefore determined by assigning the sample to the class obtaining the largest number of predictions amongst the base classifiers.

Since MV only relies on the binary outcome, in cases where an even number of data types are combined, it is possible that tied decisions may occur, in which case, they must be broken using any arbitrary heuristic provided it is chosen *a priori*. In the current investigation MV was therefore not performed for data combined from two modalities, because in such cases ties are very likely. Thus, the final classification is likely to be strongly influenced by the heuristic chosen.

### Data used for SVM integration

Alterations in grey matter (GM), white matter (WM), and neurofunction represent some of the most robust indices of individuals in the early stages psychosis (Fusar-Poli et al., 2007, 2011; Peters et al., 2010; Pettersson-Yeo et al., 2011). Furthermore, there has been increasing demand for such metrics to be used for direct clinical benefit (Matthews et al., 2006; Borgwardt and Fusar-Poli, 2012). In this context, the basis of the current investigation was to use a combinative approach specifically focusing on these neuroimaging data types. From work conducted by our own group, measures of GM, WM and neurofunction were available for 19 FEP, 19 UHR, and 23 HC subjects. To ensure that subjects were matched for age and gender for the purposes of classification, this resulted in 19, 19, and 15 FEP and HC subject pairs, UHR and HC subject pairs, and FEP and UHR subject pairs, respectively (see Table 2 for a detailed characterization of subject groups; for full details of how these data were acquired, we refer the reader to Pettersson-Yeo et al., 2013). In brief, these data were obtained as follows: (i) GM images with a 1.5 mm^3^ isotropic resolution and registered to MNI space were created using T1-weighted structural scans preprocessed using the unified segmentation procedure in conjunction with a fast diffeomorphic image registration algorithm (DARTEL), and an additional modulation step conserving the total amount of GM in each voxel after registration (Ashburner and Friston, 2005; Ashburner, 2007); implemented in SPM8 (http://www.fil.ion.ucl.ac.uk) and running under Matlab 7.1 (MathWorks, USA). As a final step, images were smoothed using a 6 mm full-width-half-maximum (FWHM) isotropic Gaussian kernel (Ashburner and Friston, 2009); (ii) for measures of WM, fractional anisotropy (FA) “skeletons” were used. These were generated from DTI data which was first preprocessed using ExploreDTI (Leemans et al., 2009) software including the RESTORE algorithm (Chang et al., 2005) to create FA maps corrected for eddy current distortion, head motion, b-matrix reorientation, and rejection of data outliers. These maps were then entered into the software package Tract Based Spatial Statistics (TBSS) (Smith et al., 2006) to create FA “skeletons” depicting each subject's unique WM network and associated FA value defined integrity for each voxel; (iii) the fMRI contrast images used were generated from an fMRI adapted Hayling sentence completion task. This involved subjects being shown sentence stems with the last word missing, for which they had to generate an overt response with a word that either made sense (i.e., Initiation), or no sense (i.e., Suppression), with the preceding stem. Blocks of five trials were interspersed with a rest condition following an ABABAB block design, in which subjects were presented with the word “REST” which they were instructed to repeat aloud, followed by a visual fixation cross. Functional images were preprocessed using SPM8 software (http://www.fil.ion.ucl.ac.uk) running under Matlab 7.1 (MathWorks, USA). Following the standard SPM8 functional imaging pipeline for preprocessing and analysis, using the parameter estimates obtained from the task's six experimental conditions: (1) generation of an overt verbal initiation response (In); (2) generation of an overt verbal suppression response (Su); (3) repetition of “REST” during the initiation condition (RI); (4) repetition of “REST” during the suppression condition (RS); (5) visual cross-fixation during the initiation condition (CFI); (6) visual cross-fixation during the suppression condition (CFS), five contrasts of interest were computed, namely, Su > In, Su > RS, In > RI, Su > CFS, In > CFI. Of the five tested in the previous work, the two primary fMRI contrasts selected for inclusion here were chosen on the basis that the conditions being contrasted represent the most cognitively divergent of the five available, and were therefore most likely to result in the greatest activation differences. These were, (i) generation of an overt verbal initiation response > visual cross-fixation during the initiation condition (In > CFI), and (ii) generation of an overt verbal suppression response > visual cross-fixation during the suppression condition (Su > CFS) (see Pettersson-Yeo et al., 2013 for more detail). For completeness, however, integrated classification was also performed by combining all seven kernels available from the previous study (i.e., GM, WM plus the five fMRI contrasts), allowing us to investigate the impact which integrating greater kernel numbers has on classification accuracy.

**Table 2:** **Demographic data for each SVM diagnostic comparison: Mean (*SD*)**.

**Characteristic**	**UHR vs. HC**			**FEP vs. HC**			**FEP vs. UHR**		
	**HC (*n* = 19)**	**UHR (*n* = 19)**	**Analysis**	**HC (*n* = 19)**	**FEP (*n* = 19)**	**Analysis**	**UHR (*n* = 15)**	**FEP (*n* = 15)**	**Analysis**
Age (years)	23.32 (3.43)	22.42 (3.42)		24.89 (4.41)	24.37 (4.71)		23.20 (3.43)	23.27 (3.69)	
Gender	9M:10F	9M:10F		12M:7F	12M:7F		9M:6F	9M:6F	
WRAT estimated premorbid IQ	107.58 (10.77)	103.16 (13.14)	*t* = −1.22	108.53 (10.48)	102.74 (9.33)	*t* = −1.68	104.87 (11.98)	103.80 (9.97)	*t* = −0.25
			*p* = 0.237			*p* = 0.110			*p* = 0.807
PANSS total^a^		52.53 (9.28)			54.37 (15.13)		53.73 (9.11)	51.80 (12.46)	*t* = −0.46
									*p* = 0.655
PANSS positive^a^		12.84 (3.67)			12.58 (3.96)		12.80 (3.65)	12.07 (3.08)	*t* = −0.60
									*p* = 0.556
PANSS negative^a^		14.00 (4.08)			13.79 (5.26)		14.33 (4.05)	13.47 (5.05)	*t* = −0.51
									*p* = 0.618
PANSS general^a^		25.68 (5.01)			28.00 (8.35)		26.60 (4.97)	26.27 (7.35)	*t* = −0.14
									*p* = 0.893
Total medication^b^		4538.49 (19226.27)			32828.58 (29788.57)		5748.75 (21628.90)	31291.57 (27178.41)	
Mean medication/day^c^		13.27 (44.43)			204.08 (116.35)		16.81 (49.75)	211.70 (109.89)	

a Symptom profile recorded at time of scan,

b Total Medication refers to the average absolute amount of medication taken by that group in standardized mg units of Chlorpromazine ± 1SD,

c *Mean Medication/day is the average medication dosage taken by each subject during their period of treatment in standardized mg units of Chlorpromazine ± 1SD. UHR, Ultra-High Risk; FEP, first episode psychosis; HC, healthy subjects; M, males; F, females; WRAT, wide range achievement test; PANSS, positive and negative syndrome scale*.

For each modality, GM, fMRI, and DTI, all voxels within each subject's image were used as features for SVM, with a whole brain mask used to remove any voxels outside of the brain area.

### SVM integration: an empirical comparison

In order to measure the relative ability of each technique to increase classification accuracy based on the integration of data from different modalities overall, a non-parametric McNemar's test was performed comparing the integrated accuracies achieved by each method against every other method, collapsed across binary diagnostic comparisons for two-way, three-way, and all data kernels combined. The results of these tests are presented in **Figures 3–5** alongside graphic visualizations showing the relative difference between the classification accuracy achieved by each integrative method and the BSMCA, for each diagnostic contrast, for combinations of two (**Figure 3**) and three (**Figure 4**) data types, in addition to all seven available kernels (**Figure 5**).

McNemar's tests with Holm-Bonferroni correction (Holm, 1979) were also performed comparing subject classifications of each individual integrated classifier vs. those of the corresponding BSMCA for each contrast, in order to identify if any individual observed difference was statistically significant.

### Integrated classification accuracies

**Figure 2:**
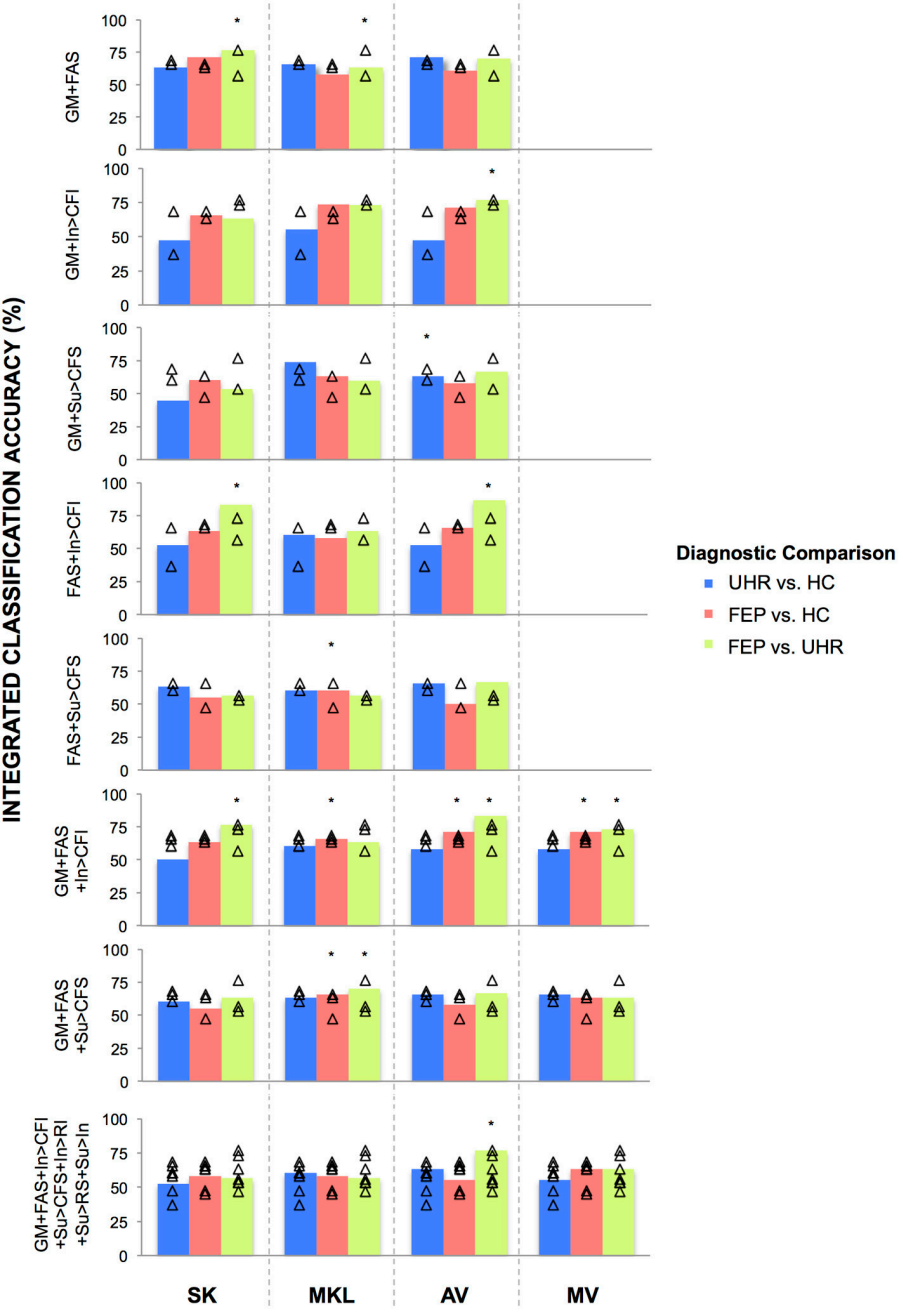
**Classifications accuracies achieved for discriminating FEP, UHR, and HC subjects, by integrating data in two-, three-, and seven-way combinations, using SK, MKL, AV, or MV**. SK, Un-weighted simple sum of kernels; MKL, Multi-Kernel Learning; AV, Prediction Averaging; MV, Majority Voting; GM, grey matter; FAS, fractional anisotropy skeleton; In, generation of an overt verbal initiation response; Su, generation of an overt verbal suppression response; RI, repetition of “REST” during the initiation condition; RS, repetition of “REST” during the suppression condition; CFI, visual cross-fixation during the initiation condition; CFS, visual cross-fixation during the suppression condition. ^*^Integrated classification accuracy significant at *p* < 0.05 FWE corrected. ^△^Single modality classification accuracies of the base kernels being integrated.

Whilst the primary aim of the study was to investigate the relative ability of different integrative methods to enhance classification accuracy relative to the BSMCA, permutation tests were also conducted to examine the significance of the each integrated classification accuracy relative to chance accuracy. First, generalizability was tested using LOOCV. Subjects were then randomly assigned to a class and the LOOCV cycle repeated 1000 times. This provided a distribution of accuracies reflecting the null hypothesis that the integrated classifier did not exceed chance. The number of times where the permuted accuracy was greater than or equal to the true accuracy was then divided by 1000 to estimate a *p*-value. In order to correct for multiple comparisons, a Holm-Bonferroni step down procedure (Holm, 1979) was employed (see Figure 2).

## Results

**Figure 3:**
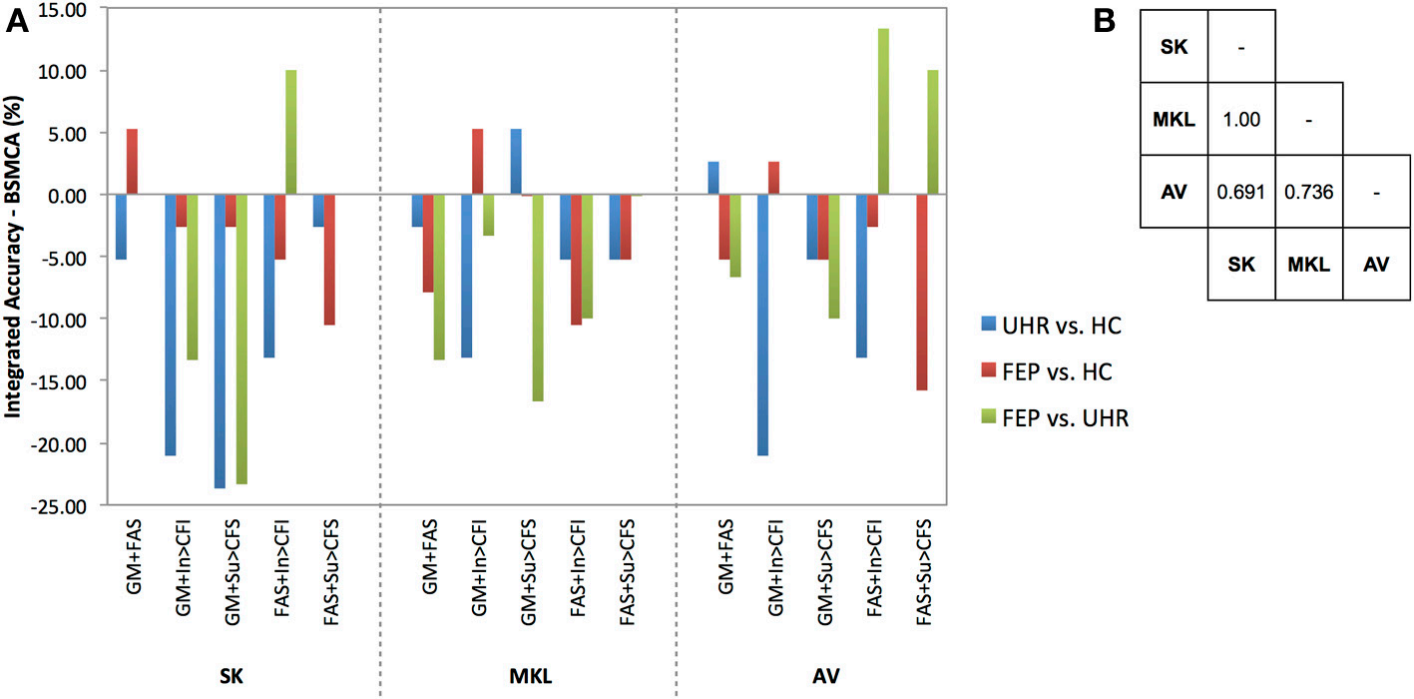
**(A)** Difference between the integrated accuracy achieved using SK, MKL, or AV, and the BSMCA, discriminating UHR and FEP subjects from HCs, and each other, using two-way combinations of sMRI, DTI, and fMRI data. **(B)** Results of McNemar's tests comparing subject classifications achieved by each integrative method collapsed across SVM contrasts and data combinations. SK, Un-weighted simple sum of kernels; MKL, Multi-Kernel Learning; AV, Prediction Averaging; GM, grey matter; FAS, fractional anisotropy skeleton; In, generation of an overt verbal initiation response; Su, generation of an overt verbal suppression response; RI, repetition of “REST” during the initiation condition; RS, repetition of “REST” during the suppression condition; CFI, visual cross-fixation during the initiation condition; CFS, visual cross-fixation during the suppression condition.

**Figure 4:**
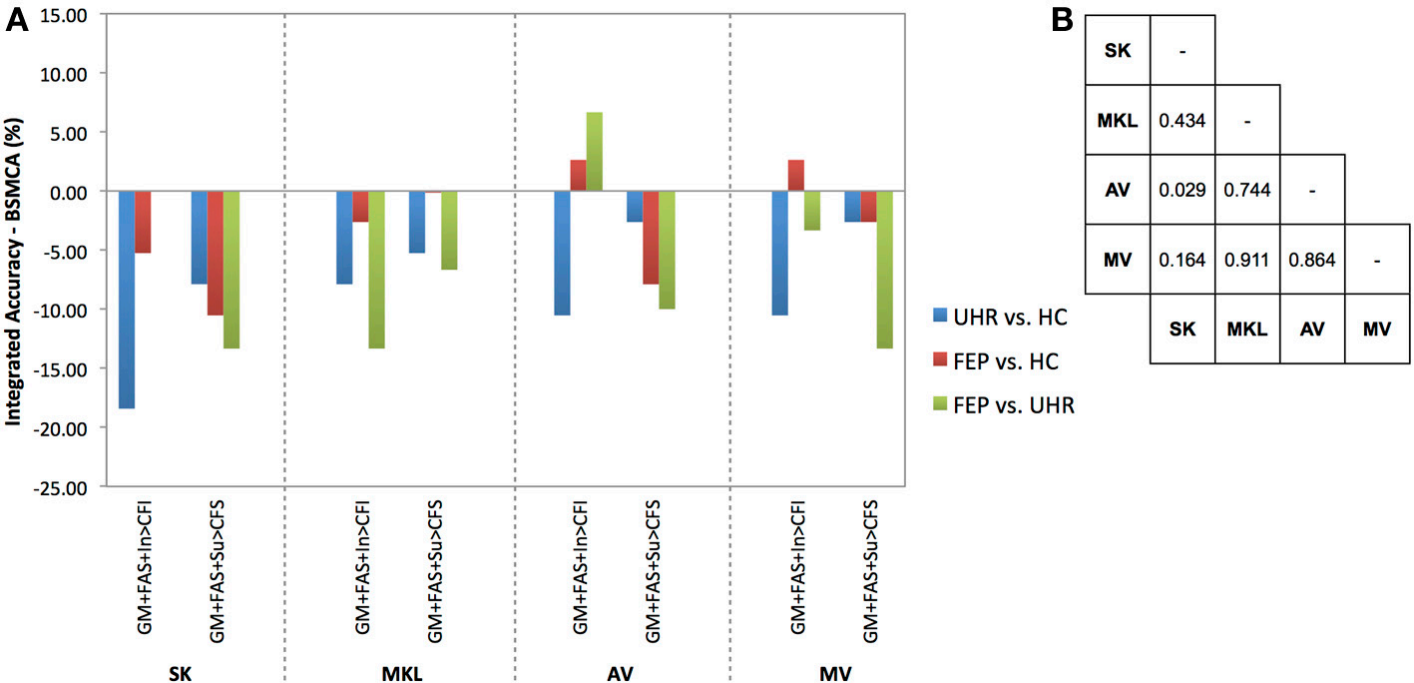
**(A)** Difference between the integrated accuracy achieved using SK, MKL, AV, or MV, and the BSMCA, discriminating UHR and FEP subjects from HCs, and each other, using three-way combinations of sMRI, DTI, and fMRI data. **(B)** Results of McNemar's tests comparing subject classifications achieved by each integrative method collapsed across SVM contrasts and data combinations. SK, Un-weighted simple sum of kernels; MKL, Multi-Kernel Learning; AV, Prediction Averaging; MV, Majority Voting; GM, grey matter; FAS, fractional anisotropy skeleton; In, generation of an overt verbal initiation response; Su, generation of an overt verbal suppression response; RI, repetition of “REST” during the initiation condition; RS, repetition of “REST” during the suppression condition; CFI, visual cross-fixation during the initiation condition; CFS, visual cross-fixation during the suppression condition.

**Figure 5:**
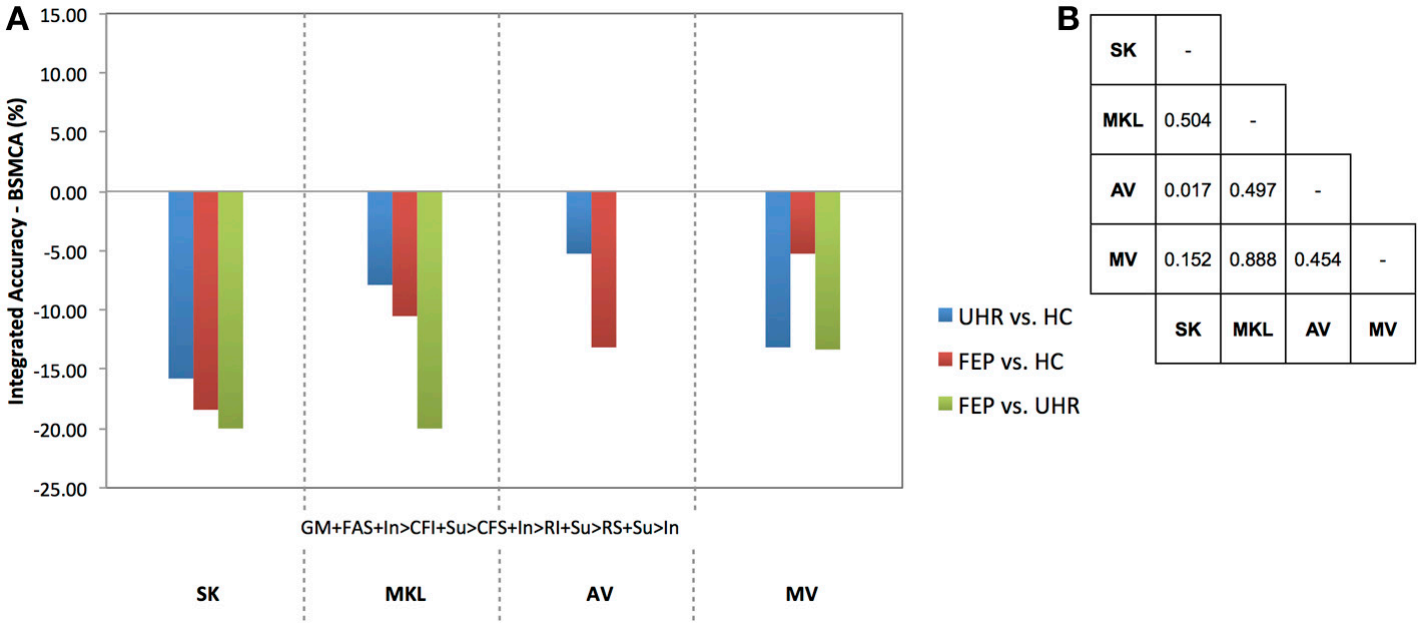
**(A)** Difference between the integrated accuracy achieved using SK, MKL, AV, or MV, and the BSMCA, discriminating UHR and FEP subjects from HCs, and each other, using seven-way combinations of sMRI, DTI, and fMRI data. **(B)** Results of McNemar's tests comparing subject classifications achieved by each integrative method collapsed across SVM contrasts and data combinations. SK, Un-weighted simple sum of kernels; MKL, Multi-Kernel Learning; AV, Prediction Averaging; MV, Majority Voting; GM, grey matter; FAS, fractional anisotropy skeleton; In, generation of an overt verbal initiation response; Su, generation of an overt verbal suppression response; RI, repetition of “REST” during the initiation condition; RS, repetition of “REST” during the suppression condition; CFI, visual cross-fixation during the initiation condition; CFS, visual cross-fixation during the suppression condition.

In a substantial majority of comparisons across two, three, and seven kernel combinations, the BSMCA was higher than any of the classifier combination methods evaluated (see Figures 3–5 and supplementary material). The minority of comparisons for which individual classifier combination methods produced higher accuracy than the BSMCA is reported below.

### Un-weighted sum of kernels

#### Data integrated from two modalities

Using SK, the ability of sMRI and DTI data combined to differentiate FEP from HC subjects was increased to 71.05% representing an approximate increase of 6% relative to the BSMCA. Furthermore, combining DTI and fMRI data using an SK approach, it was possible to discriminate FEP from UHR subjects with 83.33% accuracy representing an approximate increase of 10% relative to the BSMCA (Figures 2, 3).

#### Data integrated from three modalities

By combining three different data types using SK it was not possible to increase classification accuracy relative to the BSCMA for any of the diagnostic comparisons (see Figure 4).

#### Data integrated from seven kernels across three modalities

Based on the integration of seven kernels encompassing three data types, SK was unable to increase classification accuracy for any of the diagnostic comparisons relative to the BSMCA (see Figure 5).

### Multi kernel learning

#### Data integrated from two modalities

Using MKL, the ability of sMRI and fMRI data combined to differentiate FEP from HC subjects, and UHR from HC subjects, was increased to 73.68% subjects representing for both comparisons an approximate increase of 5% relative to the BSMCA (see Figures 2, 3).

#### Data integrated from three modalities

Based on the integration of three data types, MKL was unable to increase classification accuracy for any of the diagnostic comparisons relative to the BSMCA (see Figure 4).

#### Data integrated from seven kernels across three modalities

Based on the integration of seven kernels encompassing three data types, MKL was unable to increase classification accuracy for any of the diagnostic comparisons relative to the BSMCA (see Figure 5).

### Prediction averaging

#### Data integrated from two modalities

Using AV, the ability of sMRI and DTI data combined to differentiate UHR from HC subjects was increased to 71.05% representing an approximate increase of 3% relative to the BSMCA (see Figure 3). Similarly, combining sMRI and fMRI (contrast In > CFI) data using AV enhanced classification of FEP from HC subjects to 71.05%, representing an approximate increase of 3% relative to the BSMCA. When applied to the integration of DTI with fMRI data in order to discriminate FEP from UHR subjects, AV was able to increase classification accuracy to 86.67 and 66.67%, using the In > CFI and Su > CFS contrasts, respectively, representing approximate increases of 13 and 10% relative to the BSMCA in each case (see Figures 2, 3).

#### Data integrated from three modalities

Combining sMRI, DTI, and fMRI (contrast In > CFI) data using AV, the ability to distinguish FEP from HC subjects, and FEP from UHR subjects, was increased to 71.05 and 83.33%, respectively. In each case this represented an approximate increase of 3 and 7% relative to the BSMCA (see Figures 2, 4).

#### Data integrated from seven kernels across three modalities

Based on the integration of seven kernels encompassing three data types, AV was unable to increase classification accuracy for any of the diagnostic comparisons relative to the BSMCA (see Figure 5).

### Majority voting

#### Data integrated from three modalities

Using MV, it was possible to discriminate FEP from HC subjects with 71.05% accuracy based on the three-way combination of sMRI, DTI, and fMRI (contrast In > CFI) data. This represented an approximate increase of 3% relative to the BSMCA (see Figures 2, 4).

#### Data integrated from seven kernels across three modalities

Based on the integration of seven kernels encompassing three data types, MV was unable to enhance classification accuracy for any of the diagnostic comparisons relative to the BSMCA (see Figure 5).

### An empirical comparison of methods

#### Data combined from two modalities

Collapsed across all comparisons, the results of the McNemar's tests comparing subject classifications made by each method for all two-way combinations, though not statistically significant, gave the following best-to-worst ranking of methods based on their respective *p*-values: AV, MKL, SK (see Figure 3B) (as noted, MV was not performed for data combined from two modalities due to the potential over influence of the heuristic used where ties occur). However, in terms of the greatest individual accuracy increases achieved, relative to the BSMCA, MKL was broadly outperformed by both AV and SK (see Figure 3). Across diagnostic comparisons, greater integrated accuracies were achieved for the FEP vs. UHR comparison by each of the three integrative approaches, relative to the other two diagnostic comparisons.

#### Data combined from three modalities

Collapsed across all comparisons the results of the McNemar's tests comparing subject classifications made by each method for all three-way combinations, though not all significant, gave a best-to-worst ranking of methods based on their respective *p*-values: AV, MV, MKL, and SK. Consistent with this, in terms of the greatest individual increases in classification accuracy, relative to the BSMCA, AV performed better than MV, which in turn performed better than SK and MKL—neither of which were able to increase classification accuracy above the BSMCA. Across diagnostic comparisons, consistent with the two-way combinations, the best integrated accuracies were generally achieved for the FEP vs. UHR comparison, followed by the FEP vs. HC, and then UHR vs. HC comparisons.

#### Data combined from seven kernels encompassing three modalities

Collapsed across all comparisons the results of the McNemar's tests comparing subject classifications made by each method, though not statistically significant, gave a best-to-worst ranking of methods based on their respective *p*-values: AV, MKL, MV, and SK. As shown in Figure 5, however, no integrative method was able to enhance classification accuracy relative to the BSMCA, with the best result represented by AV for the FEP vs. UHR comparison where it was only able to match the BSMCA. In terms of the smallest classification accuracy reduction relative to the BSMCA, AV performed better than MV, MV better than MKL, and MKL better than SK. Across diagnostic comparisons, consistent with the two-and three-way combinations, the best integrated accuracies (i.e., least decreasing relative to the BSMCA) were generally achieved for the FEP vs. UHR comparison, followed by the FEP vs. HC, and then UHR vs. HC comparisons.

### Integrated classification accuracies

The results of the McNemar's tests, with Holm-Bonferroni correction, comparing classification accuracies relative to the corresponding BSMCA found none of the differences—either decreases or increases—to be statistically significant (*p* > 0.05).

## Discussion

In the current study we performed an empirical comparison of the relative abilities of four distinct methodological approaches to increase the classification accuracy of SVM by combining multimodal neuroimaging data. Specifically, each method was applied to three separate diagnostic comparisons related to the early stages of psychosis, utilizing combinations of data from sMRI, fMRI, and/or DTI modalities. The most striking feature of our results is that in most cases, the BSMCA provided higher classification accuracy than any multi-modal combination method. With regard to the few contrasts that did improve, we note that, although none of the differences were statistically significant: (i) in agreement with our first hypothesis, an un-weighted simple sum of kernels appeared to perform slightly better than a relatively more sophisticated weighted multi kernel learning for integrating two modalities, (ii) inconsistent with our first hypothesis AV appeared to be slightly more effective than either SK or MKL for increasing classification accuracy across two and three modality combinations, (iii) in agreement with our second hypothesis, AV also appeared to perform slightly better than SK in terms of the number and magnitude of classification increases for integrating three modalities, (iv) contrary to our second hypothesis, MKL was unable to provide any increase in classification accuracy relative to the BSCMA, but MV was able to produce slight improvements in some cases, and (v) in agreement with our third hypothesis, we found that the performance of each approach was dependent on the diagnostic comparison to which they were applied.

Taken together the results suggest that whilst the integration of different data types can enhance classification accuracy, the frequency of such instances may be limited. As a consequence, our results suggest that for small to moderately sized clinical neuroimaging datasets, combining different imaging modalities in a data-driven manner is not a “magic bullet” to increase classification accuracy. The findings also highlight that the potential of each integrative method to enhance classification accuracy appears to be: (i) differentially suited to different diagnostic comparisons, (ii) influenced by the number of different data types being integrated, and (iii) influenced by the specific types of data being integrated.

With respect to the influence of diagnostic comparison for example, as shown by Figures 3–5, there is a distinction between each of the four integrative methods to enhance classification accuracy dependent on the comparison to which it is applied. This may be related to the degree of complementary information provided by the data modalities to discriminate each diagnostic comparison (i.e., two different classifiers which make the same predictions for the same subjects have little complementary information to add to one another).

With respect to the second factor, the assumption that greater kernel numbers will necessarily result in greater accuracy is not supported by the results here, and simply adding more data modalities may only contribute noise that impairs the ability of the SVM to discriminate classes, possibly by increasing the uncertainty with which parameters are estimated during training. Consistent with this interpretation, the integrations with greatest increase were based on data combined from two, rather than three, modalities (see Figures 3, 4), and in the event all seven available kernels were used, no integrative method was able to increase classification accuracy. This may particularly be true if the data modalities being added do not enable classes to be discriminated in isolation. Note, however, that if kernels are preselected based on their ability to discriminate classes, this must not be done based on performance on the test data.

The results also support the notion that for the sample size investigated when fewer data types are being integrated, less computationally complex techniques such as prediction averaging and a simple summing of kernels may provide comparable, if not greater, levels of integrated accuracy in comparison to more computationally complex approaches such as MKL. This is probably because MKL requires the estimation of more parameters than can practically be estimated from the small sample size we investigated. This is also consistent with Lewis et al. who reported similar findings based on their application of integrated SVM techniques to protein interaction prediction (Lewis et al., 2006). More recent studies also support this, suggesting that MKL may instead be better suited to larger sample sizes (Damoulas and Girolami, 2008). One proposed benefit of MKL that is still partially evident in the data here, however, is the explicit ability to down-regulate the weight of a “noisy” data set, whilst still utilizing its complementary information. For example, MKL was able to combine the fMRI contrast, Su > CFS, which by itself had only been able to classify UHR from HC subjects with a statistically insignificant accuracy of 60.53%, with sMRI data and enhance overall classification accuracy by approximately 5% relative to the BSMCA (see Figure 3). In comparison, using the *same* two-way data combination, the remaining integrative techniques were unable to enhance classification accuracy, possibly due to the un-weighted contribution of the fMRI contrast acting as “noisy” interference.

In addition to the number of modalities it also seems evident that the specific types of modality being integrated is a third important factor. For example, whilst the combination of DTI and fMRI data using SK and AV provided an approximate increase 10 and 13%, respectively with regard to the differentiation of FEP and UHR subjects, combining sMRI with DTI data, or the same fMRI contrast, using the same integrative methods, did not result in a similarly increased classification accuracy (see Figure 3). As above, this is probably related to the degree of complementary information each data type offers another for discriminating each contrast. For example, alterations in GM, WM, and/or neurofunction, are unlikely to proceed equally across each stage of psychosis (i.e., GM alterations may occur sooner in the psychosis timecourse than changes in functional activation). As such, different data types may classify a given subject more, or less, easily depending on their specific psychotic stage. When considering SVM as a research, and potential real-world clinical tool, therefore, it should be emphasized that the ability to classify different clinical groups with the highest accuracy will be associated with specific data types that should be clarified. In this context, it is worth noting that should different data types not have any complementary information to add to one another, it would not be possible for an integrative approach to yield an accuracy greater than the relevant BSMCA. Hence, whilst the groups used here were smaller than those used in more traditional machine learning applications (e.g., Guodong et al., 2000), we suggest that the observed results are more likely to be a reflection of the intrinsic properties of the input data used rather than sample size; the likelihood being that the different modalities combined simply had little, or no, complementary information to offer one another. Thus, it remains that an integrative approach may in fact have provided better results had more diverse types of data been combined (e.g., genetic, cognitive, neuroimaging).

In summary, our results show that whilst integration has the potential to increase classification accuracy (by up to 13% in our data), such increases represent the exception rather than the norm. Rather, these data suggest that in the majority of cases single modality classification may provide the highest accuracy, with integration predominantly resulting in a decrease relative to the BSMCA. In addition, these findings emphasize the substantial impact of a range of factors, on the ability of integration *per se* to increase classification accuracy, including: the method used, the diagnostic groups being classified, and, the number and types of data being combined.

### Limitations

The study's main limitation may be considered to be the relatively small size of the clinical and control groups used for classification, thereby limiting the generalizability of any generated classifier(s) to future psychosis subjects. Consistent with Nieuwenhuis et al. (2012), it is possible that consistently higher integrated accuracies may have been evident had a larger sample size been used; potentially by counteracting increased noise generated as a result of combining multiple modalities. Nevertheless, it remains that the sample used here is comparable with the majority of studies that, in recent years, have used SVM to discriminate between patients and controls or between different clinical groups (Orrù et al., 2012). A second limitation is the potential impact on the findings due to having more features (e.g., voxels) than samples (e.g., subjects), commonly referred to as the “curse of dimensionality.” Whilst offset here due the fact classification was performed using a linear kernel formulation—thus limiting the number of SVM parameters to be optimized to the number of samples, plus one—it remains that by using so many features the classifier is made more vulnerable to noise, a problem potentially exacerbated by combining multiple modalities.

### Conclusion

In the current study we performed an empirical comparison of four distinct approaches for increasing SVM classification accuracy by integrating data from multiple sources on a dataset of a similar size to many clinical neuroimaging studies. Following individual application to three separate diagnostic comparisons related to the early stages of psychosis, we demonstrated that the specific integrative approach used, the number of data types integrated, and also the diagnostic comparison to which they are applied, all appear to have substantial impact on the integrated accuracy achieved. Most importantly, it appears that using a single modality, single kernel classifier often provides the best results, suggesting that combining different imaging modalities in a data-driven manner is not a “magic bullet” to increase classification accuracy for moderately sized clinical data sets. It remains possible however that this conclusion is dependent on the use of neuroimaging modalities that had little, or no, complementary information to offer one another, and that the integration of more diverse types of data would have produced greater classification enhancement. We suggest that future studies ideally examine a greater variety of data types (e.g., genetic, cognitive, and neuroimaging) in order to identify the data types and combinations optimally suited to the classification of early stage psychosis.

### Conflict of interest statement

The authors declare that the research was conducted in the absence of any commercial or financial relationships that could be construed as a potential conflict of interest.
