# Supplementary material for: An empirical comparison of different approaches for combining multimodal neuroimaging data with support vector machine
Source: Front Neurosci. 2014 Jul 15;8:189. doi: 10.3389/fnins.2014.00189 (PMC4097812; doi:10.3389/fnins.2014.00189)
Supplement: Supplementary file 1 [file DataSheet1.DOCX]

**Supplementary Material**

**An empirical comparison of different approaches for combining multimodal neuroimaging data with Support Vector Machine**

William Pettersson-Yeo*, Stefania Benetti, Andre F Marquand, Richard Joules, Marco Catani, Steve C R Williams, Paul Allen, Philip McGuire, Andrea Mechelli

**Supplementary Results**

**Supplementary Table 1:** Classification accuracies combining sMRI, DTI and fMRI data in two-, and three-, way combinations using an un-weighted sum of kernels to discriminate UHR from HCs, FEP from HCs and FEP from UHR subjects.

| **Data Combination** | **Un-weighted Sum of Kernels** | | |
| --- | --- | --- | --- |
|  | **UHR x HC** | **FEP x HC** | **FEP x UHR** |
| **GM + FAS**  **(%)** | 63.16  (68.42+65.79) | 71.05 *  (63.16+65.79) | 76.67 ^a^  (76.67+56.67) |
| **GM + In>CFI (%)** | 47.37  (68.42+36.84) | 65.79  (63.16+68.42) | 63.33  (76.67+73.33) |
| **GM + Su>CFS (%)** | 44.74  (68.42+60.53) | 60.53  (63.16+47.37) | 53.33  (76.67+53.33) |
| **FAS + In>CFI (%)** | 52.63  (65.79+36.84) | 63.16  (65.79+68.42) | 83.33 ^a^*  (56.67+73.33) |
| **FAS + Su>CFS (%)** | 63.16  (65.79+60.53) | 55.26  (65.79+47.37) | 56.67  (56.67+53.33) |
| **GM + FAS + In>CFI (%)** | 50.00  (68.42+65.79+36.84) | 63.16  (63.16+65.79+68.42) | 76.67 ^a^  (76.67+56.67+73.33) |
| **GM + FAS + Su>CFS (%)** | 60.53  (68.42+65.79+60.53) | 55.26  (63.16+65.79+47.37) | 63.33  (76.67+56.67+53.33) |
| **GM + FAS + In>CFI + In>RI + Su>CFS + Su>RS + Su>In (%)** | 52.63  (68.42+65.79+36.84+57.89+60.53+60.53+47.37) | 57.90  (63.16+65.79+68.42+65.79+47.37+44.74+63.16) | 56.67  (76.67+55.67+73.33+63.33+53.33+46.67+53.33) |

sMRI: structural MRI, DTI: diffusion tensor imaging, fMRI: functional MRI, GM: grey matter, FAS: fractional anisotropy skeleton, In: Initiation, CFI: Cross-fixation during Initiation, RI: Repetition of ‘REST’ during initiation, Su: Suppression, CFS: Cross-fixation during Suppression, RS: Repetition of ‘REST’ during Suppression UHR: Ultra-High Risk, FEP: First Episode Psychosis, HC: Healthy Subjects. ^a^ p<0.05 family-wise-error corrected. Starred figures represent accuracy increases of 0-10% (*), or >10% (**), relative to the single best accuracy of the modalities used in the integration. Figures in brackets are the accuracies for the single modalities ordered as per the data combination column.

**Supplementary Table 2:** Classification accuracies combining sMRI, DTI and fMRI data in two-, and three-, way combinations using multi kernel learning to discriminate UHR from HCs, FEP from HCs and FEP from UHR subjects.

| **Data Combination** | **Multi Kernel Learning** | | |
| --- | --- | --- | --- |
|  | **UHR x HC** | **FEP x HC** | **FEP x UHR** |
| **GM + FAS**  **(%)** | 65.79  (68.42+65.79) | 57.90  (63.16+65.79) | 63.33 ^a^  (76.67+56.67) |
| **GM + In>CFI (%)** | 55.26  (68.42 + 36.84) | 73.68 *  (63.16+68.42) | 73.33  (76.67+73.33) |
| **GM + Su>CFS (%)** | 73.68 *  (68.42 + 60.53) | 63.12  (63.16+47.37) | 60.00  (76.67+53.33) |
| **FAS + In>CFI (%)** | 60.53  (65.79+36.84) | 57.90  (65.79+68.42) | 63.33  (56.67+73.33) |
| **FAS + Su>CFS (%)** | 60.53  (65.79+60.53) | 60.53 ^a^  (65.79+47.37) | 56.67  (56.67+53.33) |
| **GM + FAS + In>CFI (%)** | 60.53  (68.42+65.79+36.84) | 65.79 ^a^  (63.16+65.79+68.42) | 63.33  (76.67+56.67+73.33) |
| **GM + FAS + Su>CFS (%)** | 63.16  (68.42+65.79+60.53) | 65.79 ^a^  (63.16+65.79+47.37) | 70.00 ^a^  (76.67+56.67+53.33) |
| **GM + FAS + In>CFI + In>RI + Su>CFS + Su>RS + Su>In (%)** | 60.53  (68.42+65.79+36.84+57.89+60.53+60.53+47.37) | 57.90  (63.16+65.79+68.42+65.79+47.37+44.74+63.16) | 56.67  (76.67+55.67+73.33+63.33+53.33+46.67+53.33) |

sMRI: structural MRI, DTI: diffusion tensor imaging, fMRI: functional MRI, GM: grey matter, FAS: fractional anisotropy skeleton, In: Initiation, CFI: Cross-fixation during Initiation, RI: Repetition of ‘REST’ during Initiation, Su: Suppression, CFS: Cross-fixation during Suppression, RS: Repetition of ‘REST’ during Suppression, UHR: Ultra-High Risk, FEP: First Episode Psychosis, HC: Healthy Subjects. ^a^ *p*<0.05 family-wise-error corrected. Starred figures represent accuracy increases of 0-10% (*), or >10% (**), relative to the single best accuracy of the modalities used in the integration. Figures in brackets are the accuracies for the single modalities ordered as per the data combination column.

**Supplementary Table 3:** SVM classification accuracies combining sMRI, DTI and fMRI data in two-, and three-, way combinations using prediction averaging to discriminate UHR from HCs, FEP from HCs and FEP from UHR subjects.

| **Data Combination** | **Prediction Averaging** | | |
| --- | --- | --- | --- |
|  | **UHR x HC** | **FEP x HC** | **FEP x UHR** |
| **GM + FAS**  **(%)** | 71.05 *  (68.42 + 65.79) | 60.53  (63.16+65.79) | 70.00  (76.67+56.67) |
| **GM + In>CFI (%)** | 47.37  (68.42 + 36.84) | 71.05 *  (63.16+68.42) | 76.67 ^a^  (76.67+73.33) |
| **GM + Su>CFS (%)** | 63.16 ^a^  (68.42 + 60.53) | 57.89  (63.16+47.37) | 66.67  (76.67+53.33) |
| **FAS + In>CFI (%)** | 52.63  (65.79+36.84) | 65.79  (65.79+68.42) | 86.67 ^a^**  (56.67+73.33) |
| **FAS + Su>CFS (%)** | 65.79  (65.79+60.53) | 50.00  (65.79+47.37) | 66.67 *  (56.67+53.33) |
| **GM + FAS + In>CFI (%)** | 57.89  (68.42+65.79+36.84) | 71.05 ^a^*  (63.16+65.79+68.42) | 83.33 ^a^*  (76.67+56.67+73.33) |
| **GM + FAS + Su>CFS (%)** | 65.79  (68.42+65.79+60.53) | 57.89  (63.16+65.79+47.37) | 66.67  (76.67+56.67+53.33) |
| **GM + FAS + In>CFI + In>RI + Su>CFS + Su>RS + Su>In (%)** | 63.16  (68.42+65.79+36.84+57.89+60.53+60.53+47.37) | 55.26  (63.16+65.79+68.42+65.79+47.37+44.74+63.16) | 76.67 ^a^  (76.67+55.67+73.33+63.33+53.33+46.67+53.33) |

sMRI: structural MRI, DTI: diffusion tensor imaging, fMRI: functional MRI, GM: grey matter, FAS: fractional anisotropy skeleton, In: Initiation, CFI: Cross-fixation during Initiation, RI: Repetition of ‘REST’ during Initiation, Su: Suppression, CFS: Cross-fixation during Suppression, RS: Repetition of ‘REST’ during Suppression. UHR: Ultra-High Risk, FEP: First Episode Psychosis, HC: Healthy Subjects. ^a^ p<0.05 family-wise-error corrected. Starred figures represent accuracy increases of 0-10% (*), or >10% (**), relative to the single best accuracy of the modalities used in the integration. Figures in brackets are the accuracies for the single modalities ordered as per the data combination column.

**Supplementary Table 4:** SVM classification accuracies combining sMRI, DTI and fMRI data in a three way combination using majority voting to discriminate UHR from HCs, FEP from HCs and FEP from UHR subjects.

| **Data Combination** | **Majority Voting** | | |
| --- | --- | --- | --- |
|  | **UHR x HC** | **FEP x HC** | **FEP x UHR** |
| **GM + FAS + In>CFI (%)** | 57.89  (68.42+65.79+36.84) | 71.05 ^a^*  (63.16+65.79+68.42) | 73.33 ^a^  (76.67+56.67+73.33) |
| **GM + FAS + Su>CFS (%)** | 65.79  (68.42+65.79+60.53) | 63.16  (63.16+65.79+47.37) | 63.33  (76.67+56.67+53.33) |
| **GM + FAS + In>CFI + In>RI + Su>CFS + Su>RS + Su>In (%)** | 55.26  (68.42+65.79+36.84+57.89+60.53+60.53+47.37) | 63.16  (63.16+65.79+68.42+65.79+47.37+44.74+63.16) | 63.33  (76.67+55.67+73.33+63.33+53.33+46.67+53.33) |

sMRI: structural MRI, DTI: diffusion tensor imaging, fMRI: functional MRI, GM: grey matter, FAS: fractional anisotropy skeleton, In: Initiation, CFI: Cross-fixation during Initiation, RI: Repetition of ‘REST’ during Initiation, Su: Suppression, CFS: Cross-fixation during Suppression, RS: Repetition of ‘REST’ during Suppression, UHR: Ultra-High Risk, FEP: First Episode Psychosis, HC: Healthy Subjects. ^a^ p<0.05 family-wise-error corrected. Starred figures represent accuracy increases of 0-10% (*), or >10% (**), relative to the single best accuracy of the modalities used in the integration. Figures in brackets are the accuracies for the single modalities ordered as per the data combination column.
